# Supplementary material for: Mediation and longitudinal analysis to interpret the association between clozapine pharmacokinetics, pharmacogenomics, and absolute neutrophil count
Source: Schizophrenia (Heidelb). 2023 Oct 18;9(1):74. doi: 10.1038/s41537-023-00404-6 (PMC10585000; doi:10.1038/s41537-023-00404-6)
Supplement: Supplementary file 1 — Supplementary Note [file 41537_2023_404_MOESM1_ESM.docx]

**Supplementary Information:**

**Mediation and Longitudinal Analysis to interpret the association between clozapine pharmacokinetics, pharmacogenomics, and absolute neutrophil count**

[Supplementary Notes: Genetic Variables 3](#_Toc147564001)

[Quality Control of Genetic data 3](#_Toc147564002)

[Exploring the effects of CYP1A2 Pharmacogenomic Variation on Neutrophil Counts in Clozapine Users 3](#_Toc147564003)

[Assessing the Impact of HLA Genotypes on Neutrophil Counts 4](#_Toc147564004)

[Supplementary Notes: Statistical Methodology 5](#_Toc147564005)

[Exclusion of the Metabolic Ratio Variable 5](#_Toc147564006)

[R Packages 5](#_Toc147564007)

[Deriving Residuals for use in Structural Equational Modelling 5](#_Toc147564008)

[Supplementary Notes: Secondary Analyses 6](#_Toc147564009)

[Characterising CYP1A2 Activity Score 6](#_Toc147564010)

[Extending the Linear Mixed Models with Genetic Covariates 6](#_Toc147564011)

[Controlling for CYP1A2 Activity in the Mediation Analysis 6](#_Toc147564012)

[Non-Residualised SEM for Mediation Analysis 7](#_Toc147564013)

[Single Variable Mediation Analyses 7](#_Toc147564014)

[Replicating Past Studies with Linear Models. 7](#_Toc147564015)

[Supplementary Figures 8](#_Toc147564016)

[Supplementary Figure 1 – Distribution of CYP1A2 Activity Scores in CLOZUK3 8](#_Toc147564017)

[Supplementary Figure 2 – Directed Acyclic Graph (DAG) showing causal paths between Clozapine Dose and Absolute Neutrophil Count. 9](#_Toc147564018)

[Supplementary Figure 3 – Comparison of Mediation Analysis Effect Sizes with and without including Genetic data. 10](#_Toc147564019)

[Supplementary Figure 4 – Results of Mediation Analysis using SEM with non-residualised variables. 11](#_Toc147564020)

[Supplementary Figure 5 – Comparison of Covariate Effect Sizes from Linear and Linear Mixed Effect Models on CLOZUK3 with Linear Model on CLOZUK2. 12](#_Toc147564021)

[Supplementary Tables 13](#_Toc147564022)

[Supplementary Table 1 – CLOZUK3 CYP1A2 PGx star allele information 13](#_Toc147564023)

[Supplementary Table 2 – CLOZUK3 HLA allele information 14](#_Toc147564024)

[Supplementary Table 3a – Directed Acyclic Graph nodes 15](#_Toc147564025)

[Supplementary Table 3b – Directed Acyclic Graph paths 16](#_Toc147564026)

[Supplementary Table 4 – Characterising CYP1A2 Activity Score 20](#_Toc147564027)

[Supplementary Table 5 – CYP1A2 Activity Score + rs2472297 regression output 21](#_Toc147564028)

[Supplementary Table 6 – Duffy Null and PGS regression output 22](#_Toc147564029)

[Supplementary Table 7 – Condensed HLA allele regression output 23](#_Toc147564030)

[Supplementary Table 8 – Single Mediator analysis of Clozapine concentration 26](#_Toc147564031)

[Supplementary Table 9 – Single Mediator analysis of Norclozapine concentration 27](#_Toc147564032)

[Supplementary Table 10 – Replication regression outputs 28](#_Toc147564033)

[Supplementary References 29](#_Toc147564034)

## Supplementary Notes: Genetic Variables

### Quality Control of Genetic data

A subset of the CLOZUK3 sample was genotyped using the Illumina Infinium Global Screening-24 (Illumina Inc., USA) at Icahn School of Medicine at Mount Sinai (New York City, USA). The “GenotypeQCtoHRC” module of the DRAGON-data pipeline was used to process the genotype data^1^. This includes quality control of calls, removing individuals with genotyping rates <0.95, and SNPs with call rates <0.95, minor allele frequency <0.01, and with Hardy-Weinberg Equilibrium mid-*p* < 10^-6^. Imputation and statistical phasing were carried out via the Michigan Imputation Server and minimac4 using the Haplotype Reference Consortium (HRC) panel^2,3^. Further quality control was performed on the imputed genotypes, described in previous work^4^. Genetic data, including Polygenic Scores (PGS) for clozapine and norclozapine metabolism, key pharmacogenomic SNPs, and CYP1A2 activity scores, were merged with the combined CLOZUK3 dataset containing FBC and PK information resulting in a dataset of 523 individuals and 1,586 assays.

### Exploring the effects of CYP1A2 Pharmacogenomic Variation on Neutrophil Counts in Clozapine Users

CYP1A2 plays a key role in clozapine metabolism, therefore it is likely that effects of CYP1A2 pharmacogenomic variability might be observed on neutrophil counts. Pharmacogenomic star alleles for *CYP1A2* were called using PyPGx v0.20.0^5^ in Python v3.9.2^6^. The imputed genotyping data for CLOZUK3 was passed through the run-chip-pipeline command to derive PGx star alleles for this pharmacogene.

As CPIC-validated information regarding CYP1A2 metabolism phenotypes and activity scores is currently not available, each haplotype was assigned an activity score in line with past work^7,8^ as described in Supplementary Table 1. These were summed to produce an overall activity score per participant, in which higher scores are reflective of increased enzyme function. Several participants were called with *CYP1A2**1F/1C alleles in the same haplotype. These alleles have opposing functions, therefore in line with past work^9^, we have treated this haplotype as a normal function allele. Pharmacogenomic variation in *CYP3A4* was not explored despite its key role in clozapine metabolism on account of the rarity of pharmacogenomic star alleles conferring non-normal function^10^, and likely difficulties that would arise when trying to fit models using such small sample sizes. Likewise, *CYP2D6* variation was not explored as many of the increased function *CYP2D6* pharmacogenomic alleles arise from structural copy number variation that cannot be reliably identified through genotyping arrays^11^.

The distribution of activity scores is shown in Supplementary Figure 1 and observed allele frequencies compared to previous research in Supplementary Table 1^12,13^. Past work has demonstrated associations between phenoconversion-corrected CYP1A2 activity score with dose-adjusted clozapine levels^8^. Thus, regression analyses were performed to determine whether uncorrected CYP1A2 activity score was associated with dose-adjusted clozapine and norclozapine levels in our sample. The *glm()* function was used with a gamma distribution and ‘log’ link function. Plasma clozapine concentration was included as the outcome variable alongside the standardised predictors CYP1A2 activity score, daily clozapine dose, age, age^2^, sex, and TDS; the same was repeated with plasma norclozapine concentration as the outcome variable.

For the primary analysis, estimated CYP1A2 activity scores were included in a linear mixed-effect model alongside medication variables (i.e., daily dose, plasma clozapine and norclozapine concentration), pharmacogenomic SNPs, and other covariates (i.e., age, age^2^, sex, TDS) to determine their impact on ANC.

Finally, two further models were fit to compare the effect size of CYP1A2 activity score with, and without including the intergenic *CYP1A1-CYP1A2* SNP, rs2472297, in the model. There is evidence that this SNP both regulates CYP1A2 activity^14^ and is in weak linkage disequilibrium with the *1F allele. Therefore, it was added as a covariate in a second model to determine whether its inclusion impacts any association between measures of CYP1A2 activity with ANC in our sample.

### Assessing the Impact of HLA Genotypes on Neutrophil Counts

Past research has observed associations between HLA genotypes with risk for clozapine-induced agranulocytosis^15–17^. Therefore, we explored whether these alleles were associated with ANC in the CLOZUK3 sample. Pre-imputation array data was restricted to chromosome 6 using PLINK v1.9^18,19^. HIBAG v1.34.1^20^ was used to derive HLA alleles, alongside the pre-built InfiniumGlobal-European-HLA4-hg19 prediction model, which best fits the CLOZUK3 data concerning both the genotyping platform and the sample ancestry. This allowed imputation of available HLA genes (-A, -B, -C, -DPB1, -DQA1, -DQB1, -DRB1) to four-digit alleles.

After imputation, alleles with a MAF > 1% were retained for further analysis (Supplementary Table 2), and linear mixed-effect models were used to explore their impact on ANC. Following Levin et al. (2015), genotypes were weighted by posterior imputation probabilities to derive their estimated allele dosage. These were merged with the FBC/PK dataset so that HLA genotypes were associated with the longitudinal assay data (N = 540; 1,627 assays). Each HLA allele was included as a covariate alongside daily clozapine dose, plasma clozapine and norclozapine concentrations, TDS, sex, age, and age^2^. Covariates were standardised and regressed against ANC, with participant ID included as a random intercept term. A total of 116 alleles across 7 HLA genes were included in the regression analyses. Correction for multiple comparisons was performed using the False Discovery Rate (FDR) and Bonferroni correction.

## Supplementary Notes: Statistical Methodology

### Exclusion of the Metabolic Ratio Variable

Previous work demonstrated that including the clozapine/norclozapine ratio (“metabolic ratio”) as a covariate in a regression model of ANC considerably attenuated the effect sizes of both clozapine and norclozapine plasma concentrations ^22^. However, incorporating ratio variables in regression models has been criticised on the basis that they may result in deceptive statistical artifacts ^23^. Equally, it is possible that the metabolic ratio acts as a collider variable (Supplementary Figure 2; Supplementary Tables 3a and 3b). Controlling for collider variables through their inclusion as covariates, may also introduce spurious associations as described in the causal inference literature ^24^. Therefore, the decision was made to exclude metabolic ratio from the present analyses.

### R Packages

The full CLOZUK3 dataset was used for linear mixed-effect regression models and single-mediator analyses, benefitting from multiple FBC/pharmacokinetic measurements per patient over time. *lme4* and *lmerTest* were used to fit the LMMs^25,26^. The *mediation* package^27^ was used to perform single-mediation analyses in the longitudinal data.

The CLOZUK3 dataset was transformed into cross-sectional data by taking the lowest observation of ANC for each participant. This reduced dataset was used for multiple-, and single-mediator analyses using Structural Equation Modelling (SEM) in *lavaan*^28^, and in replication analyses (i.e., Linear Models) of previous research^22,29^. Before inclusion in all regression and structural equation models, covariates were standardised (mean-centred and scaled) using the *datawizard* R package^30^.

### Deriving Residuals for use in Structural Equational Modelling

To account for covariates in the mediation analysis, residualised variables were incorporated during SEM. Each variable was fit as an outcome in separate regression analyses, with the covariates, age, age^2^, sex, and TDS. ANC was included as the outcome in a linear model. Daily clozapine dose was log-transformed and included as an outcome variable in a linear model. Plasma Clozapine and Norclozapine levels were included in a generalised linear model using a gamma distribution and log link function. Linear models were fit using the *lm()* function, and generalised linear models were fit using the *glm()* function. The residuals of the predictors were standardised and then used in place of their parent measure across all mediation analyses unless otherwise specified. In a further model, the impact of CYP1A2 activity score was accounted for by including it alongside age, age^2^, sex, and TDS in the regression models to produce the residualised variables.

## Supplementary Notes: Secondary Analyses

### Characterising CYP1A2 Activity Score

Two generalised linear models were performed to determine whether estimated CYP1A2 activity scores were associated with dose-adjusted plasma concentrations of clozapine and its primary metabolite, norclozapine. CYP1A2 activity score was associated with plasma clozapine concentration (β = 0.050; *p* = 0.036) and plasma norclozapine concentration (β = 0.060; *p* = 0.010) in models adjusting for daily clozapine dose, age, age^2^, sex, and TDS (Supplementary Table 4).

### Extending the Linear Mixed Models with Genetic Covariates

To assess the impact, if any, of rs2472297 on the association between CYP1A2 activity score with ANC (Supplementary Table 5), two linear mixed-effect models were fit with and without the SNP as an additional covariate in the model. CYP1A2 activity score was significantly associated with ANC (β = 0.145; *p* = 0.004). When the SNP was included as a covariate, there was negligible change in the effect size of CYP1A2 activity score (β = 0.142; *p* = 0.011), and as in the previous pharmacogenomic analysis (Table 3, Main Text), rs2472297 was not associated with ANC in this model (β = 0.004; *p* = 0.949). This indicates that the observed association between CYP1A2 activity score and ANC is likely not conflated with rs2472297.

There was no evidence of associations between Polygenic Scores for either clozapine metabolism (β = 0.034; *p* = 0.532) or norclozapine metabolism (β = -0.009; *p* = 0.863) with ANC. However, a strong negative association (β = -0.770; *p* = 0.002) between the Duffy-Null genotype and ANC was observed in the CLOZUK3 sample. The presence of the Duffy-Null genotype was linked with a decline in neutrophils of approximately 770 cells/mm^3^ in comparison to non-carriers. Full estimates from these additional genetic models are included in Supplementary Table 6.

HLA genotypes were similarly included in linear mixed-effect models to explore whether variation in the HLA region influenced neutrophil levels in clozapine users. An overview of these findings is shown in Supplementary Table 7. After controlling for multiple comparisons, no HLA alleles were significantly associated with ANC in the CLOZUK3 sample; this suggests little influence of the HLA region on neutrophil counts in these participants. Prior to this correction, 3 HLA alleles demonstrated nominally significant associations. These included *HLA-DRB1*16:01* (β = 0.087; *p* = 0.001) and *HLA-DRB1*04:04* (β = 0.073; *p* = 0.011), which were positively associated with ANC, alongside *HLA-DRB1*01:03* (β = -0.068; *p* = 0.016), which was inversely associated with ANC.

### Controlling for CYP1A2 Activity in the Mediation Analysis

Linear mixed-effect models revealed a significant association between CYP1A2 activity score and ANC in the CLOZUK3 sample. Thus, to account for the impact of this pharmacogenomic variation on clozapine metabolism, and potentially neutrophil levels, the activity score was residualised out of the included variables, as previously described. In this instance, both the association between dose and ANC (β = 0.100, *p* = 0.077), and the indirect effect via clozapine and norclozapine were weakened (β = 0.050, *p* = 0.077). While this attenuation of effects may be due to the inclusion of the CYP1A2 activity score, it could also arise because of the reduced sample size with complete genetic information. Therefore, the primary model was repeated on this subset of the CLOZUK3 sample, to determine how this smaller sample size affected the model output. This resulted in inflated estimates for the previously significant direct (β = 0.251, *p* = 0.077) and indirect effects (β = 0.125, *p* = 0.077). A comparison of the three models (Supplementary Figure 3) showed that using a reduced sample size results in larger confidence intervals indicative of a loss of statistical power. Interestingly, controlling for CYP1A2 activity score reduces this uncertainty, bringing the intervals in line with those seen in the primary model, albeit with slightly smaller effect sizes.

### Non-Residualised SEM for Mediation Analysis

SEM was also used on non-residualised versions of the variables (Supplementary Figure 4) to ensure that controlling for covariates in this way didn’t create spurious results. As in the residualised model, there was a significant positive effect of daily clozapine dose on lowest ANC (β = 0.110, *p* = 0.017). Equally, there was a significant indirect effect via both clozapine and norclozapine plasma concentrations (β = 0.054, *p* = 0.015) with no evidence for mediation by plasma clozapine concentration alone (β = - 0.017, *p* = 0.254).

### Single Variable Mediation Analyses

SEM was performed with residualised plasma clozapine concentration as the lone mediator between dose and lowest ANC (Supplementary Table 8). As before, a significant effect of dose on lowest ANC was observed (β = 0.172, *p* = 1.1 x 10^-4^), with no support for clozapine as a mediating variable (β = 0.008, *p* = 0.394). A secondary, causal mediation analysis was performed using the full CLOZUK3 dataset. These results are consistent with the findings derived from using SEM, showing a significant direct effect of daily clozapine dose (ADE = 0.155, *p* < 2 × 10^-16^) and no indirect effect transmitted via clozapine plasma concentration (ACME = 0.006, *p* = 0.574).

A complementary single-mediator SEM was performed with norclozapine as the sole mediating variable. A significant direct effect of daily clozapine dose on lowest ANC was observed (β = 0.154, *p* = 6.1 x 10^-4^). Additionally, there is some evidence of a mediating effect when we consider norclozapine plasma concentration alone (β = 0.025, *p* = 0.048). When this analysis was replicated using the longitudinal sample, a significant direct effect was observed again (ADE = 0.133, *p* < 2 × 10^-16^). Furthermore, a significant indirect effect via norclozapine was also observed (ACME = 0.029, *p* = 0.008), shown in Supplementary Table 9.

### Replicating Past Studies with Linear Models.

Three linear models were fit in-line with Willcocks et al., (2021), excluding the covariates Time on Treatment (absent in CLOZUK3) and Days between Assays (as FBC and pharmacokinetic assays were performed on the same day for all our data points), with results reported in Supplementary Table 10. A forest plot comparing standardised regression coefficients obtained from the CLOZUK3 and CLOZUK2 samples is shown in Supplementary Figure 5.

In the first model, daily dose (β = 0.126; *p* = 0.006) and norclozapine level (β = 0.208; *p* = 0.015) were both positively associated with ANC. Although not significant, clozapine was negatively associated with ANC (β = -0.121; *p* = 0.149). The addition of metabolic ratio in the second model, reduced effect sizes for both clozapine plasma concentration (β = -0.052; *p* =0.729) and norclozapine plasma concentration (β = 0.139; *p* = 0.358). Daily clozapine dose was unaffected by this (β = 0.127; *p* = 0.006), and the metabolic ratio itself was not significantly associated with ANC (β = -0.045; *p* = 0.581). In the final model, no pharmacogenomic SNPs were associated with ANC. Furthermore, the previous association between daily dose and ANC became smaller and non-significant (β = 0.073; *p* = 0.215).

## Supplementary Figures

### Supplementary Figure 1 – Distribution of CYP1A2 Activity Scores in CLOZUK3


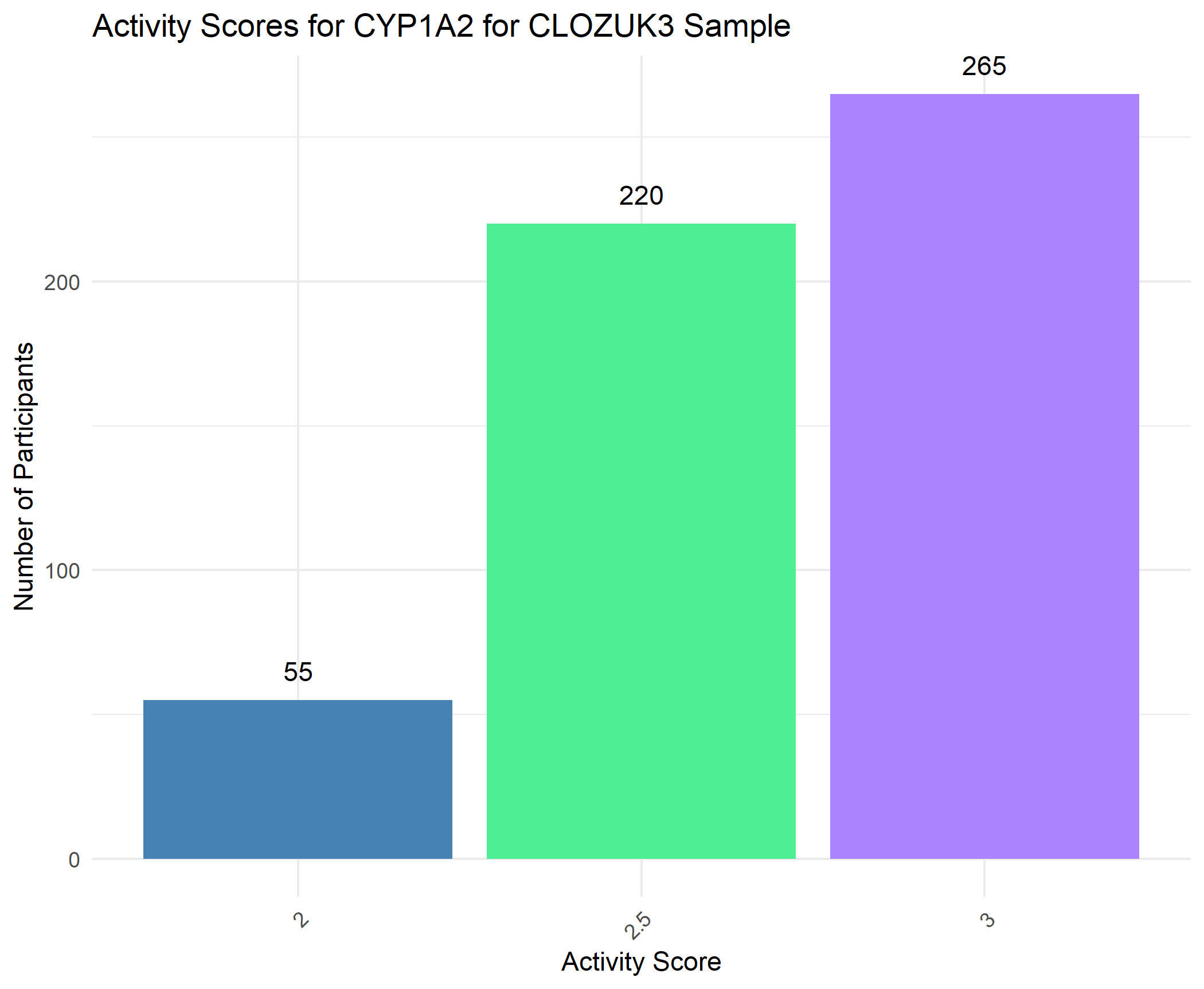


Supplementary Figure 1. Distribution of pharmacogenomic allele derived CYP1A2 activity scores observed in the CLOZUK3 sample.

###
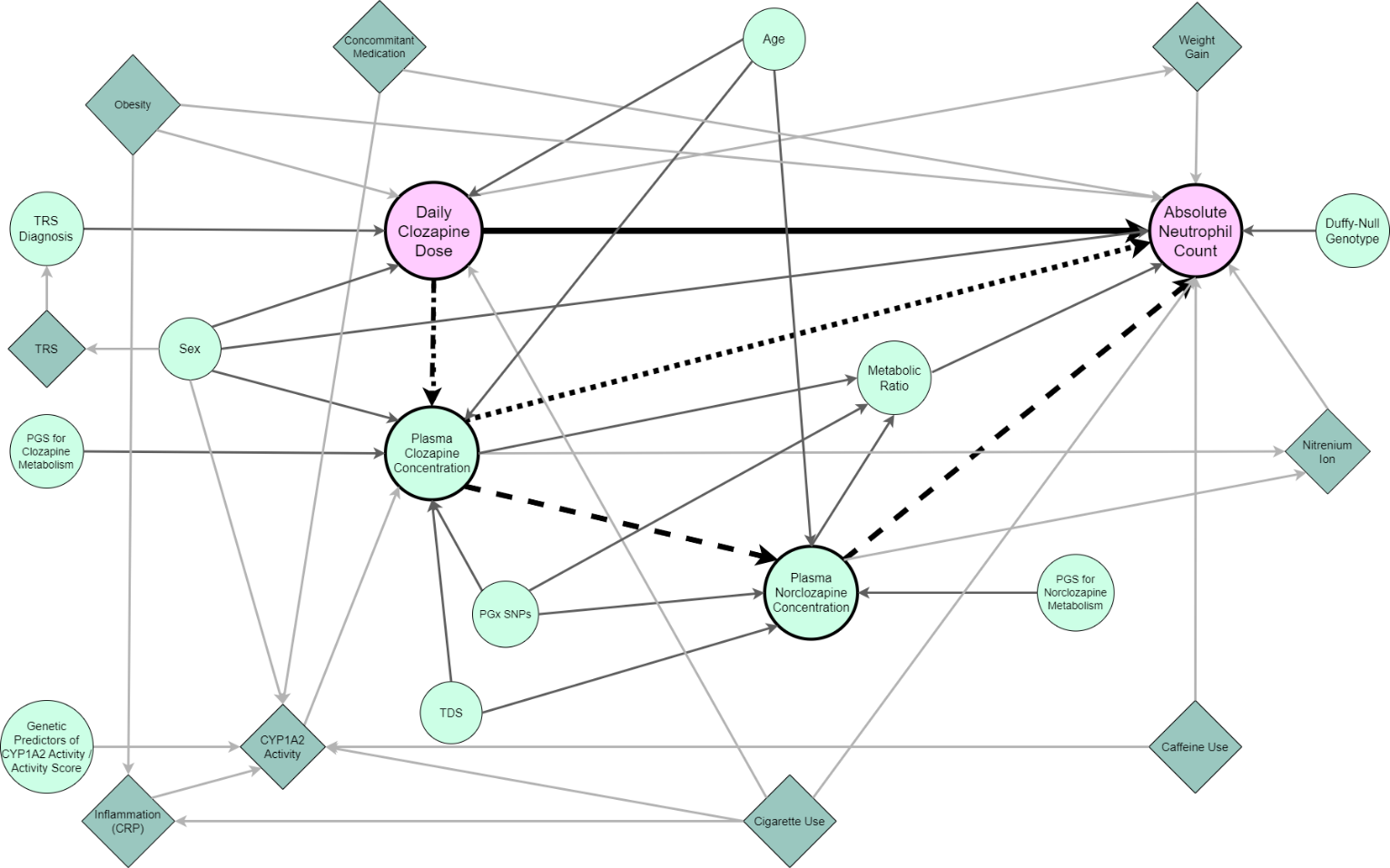
Supplementary Figure 2 – Directed Acyclic Graph (DAG) showing causal paths between Clozapine Dose and Absolute Neutrophil Count.

Supplementary Figure 2. Variables included in DAG showing the possible causal associations between clozapine dose and absolute neutrophil count. Pink circles represent the exposure (Daily Clozapine Dose) and outcome (Absolute Neutrophil Count). Light green circles represent measured variables, whereas dark green diamonds represent latent variables. Dark grey arrows represent paths between observed variables, whereas light grey arrows represent any path in which one (or both) of the variables involved are unobserved. Black paths represent effects of interest in the main regression and mediation analyses (solid = direct effect; dotted = indirect effect via mediator 1; dashed = indirect effect via mediators 1 & 2; dot-dash = path shared by both indirect effects). TRS = Treatment Resistant Schizophrenia; PGS = Polygenic Score; PGx = Pharmacogenomic; TDS= Time between Dose and Sample.

### Supplementary Figure 3 – Comparison of Mediation Analysis Effect Sizes with and without including Genetic data.


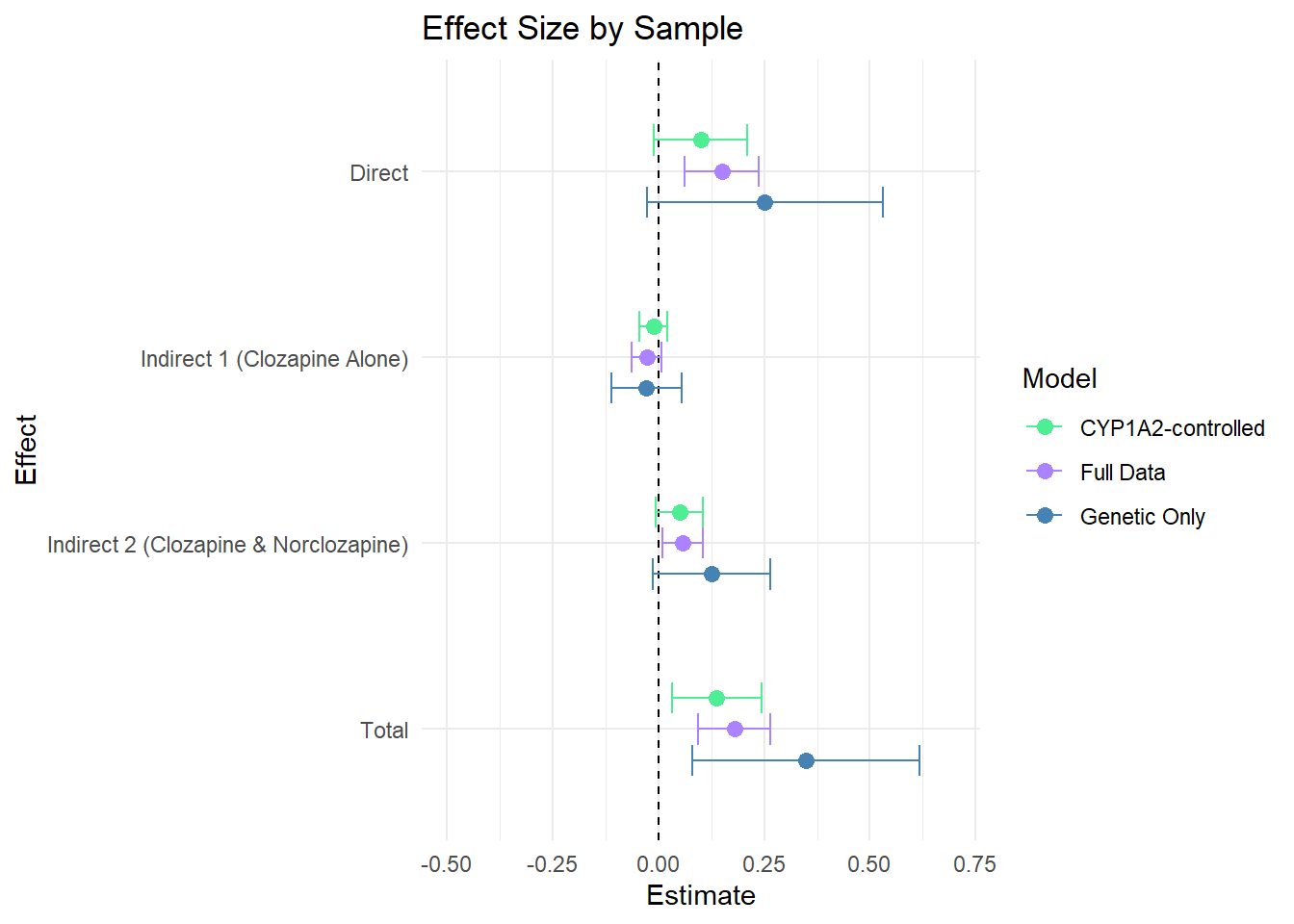


Supplementary Figure 3. Forest plot comparing effect estimates between the three Structural Equation Models. ‘Full data’ refers to the first model containing the full CLOZUK3 sample. ‘Genetic Only’ refers to the same model as in Full data, but performed on the subset of the sample for whom genetic data is available. The final model, ‘CYP1A2-controlled’, was performed on the genetic only sample but controls for CYP1A2 activity scores when creating the residualised variables. Effect estimates are standardised, and error bars show 95% confidence intervals.

### Supplementary Figure 4 – Results of Mediation Analysis using SEM with non-residualised variables.


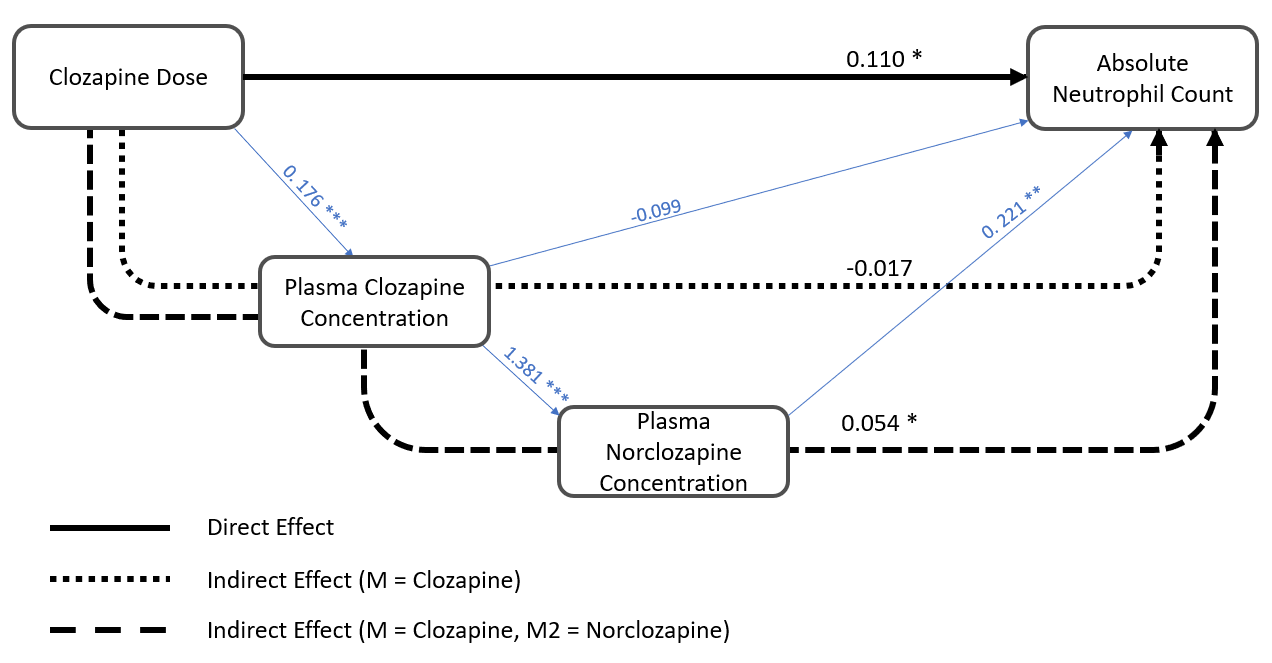


Supplementary Figure 4. Path diagram showing association between Dose and Lowest ANC with Clozapine and Norclozapine as mediators. Plot edges are labelled with standardised regression coefficients. Variables included in SEM are the parent, non-residualised variables. SEM = Structural Equation Modelling.

* p<0.05 ** p<0.01 *** p<0.001

### Supplementary Figure 5 – Comparison of Covariate Effect Sizes from Linear and Linear Mixed Effect Models on CLOZUK3 with Linear Model on CLOZUK2.


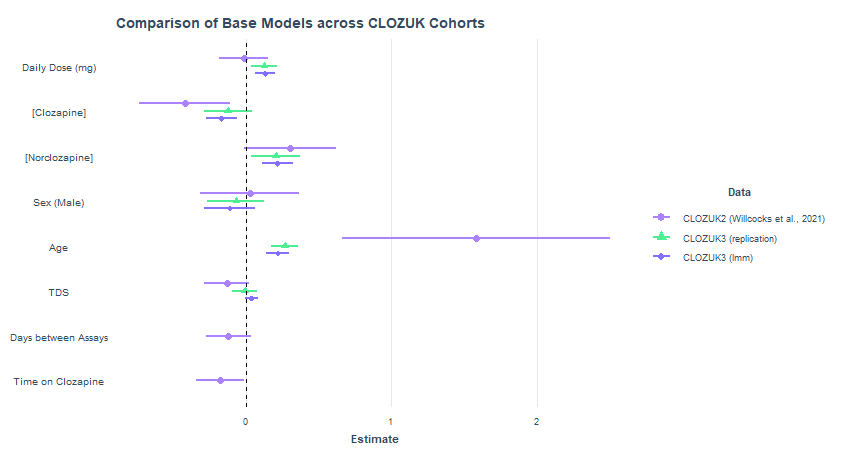


Supplementary Figure 5. Forest plot comparing standardised regression coefficients from equivalent models between CLOZUK2 (Willcocks et al., 2021) and CLOZUK3 (present work) datasets. The CLOZUK2 and CLOZUK3 (replication) models explore the association between covariates and lowest value of ANC in cross-sectional samples. The CLOZUK3 (lmm) model utilises the available longitudinal data, incorporating a random effect variable for participant ID. Error bars show 95% Confidence Intervals. ANC = Absolute Neutrophil Count; TDS = Time Between Dose and Sample; lmm = Linear Mixed-Effect Model.

## Supplementary Tables

### Supplementary Table 1 – CLOZUK3 CYP1A2 PGx star allele information

| **CYP1A2 PGx Star Allele** | **Activity Score** | **Frequency (CLOZUK3)** | **EUR Frequency**^12^ | **EUR ALFA Allele Frequency**^13^ |
| --- | --- | --- | --- | --- |
| *1A | 1 | 0.29 | 0.298 | NA |
| *1C | 0.5 | 0 | NA | 0.013 |
| *1F | 1.5 | 0.67 | 0.699 | 0.704 |
| *1C*1F | 1 | 0.041 | NA | NA |

Supplementary Table 1. CYP1A2 Pharmacogenomic (PGx) star allele activity identified in CLOZUK3. Table shows activity scores assigned to each allele, alongside a comparison of allele frequencies in samples from other populations of European ancestry. PGx = Pharmacogenomic; EUR = European.

### Supplementary Table 2 – CLOZUK3 HLA allele information

| **Gene** | **Total Alleles Identified in CLOZUK3** | **Number of Alleles included in Analysis (MAF > 1%)** | **Excluded Alleles (MAF < 1%)** |
| --- | --- | --- | --- |
| HLA-A | 28 | 17 | 02:02, 02:06, 29:01, 30:04, 33:01, 34:02, 36:01, 66:01, 69:01, 74:01, 80:01 |
| HLA-B | 49 | 25 | 07:05, 15:02, 15:03, 15:10, 15:16, 15:17, 15:18, 27:02, 35:02, 35:08, 39:06,  40:02, 40:06, 41:01, 41:02, 42:01, 42:02, 44:05, 47:01, 56:01, 39:24, 48:01, 51:08, 81:01 |
| HLA-C | 26 | 17 | 02:10, 03:02, 08:01, 15:05, 16:02, 14:03, 15:04, 16:04, 18:01 |
| HLA-DPB1 | 18 | 13 | 02:02, 09:01, 15:01, 16:01, 19:01 |
| HLA-DQA1 | 14 | 11 | 03:02, 06:01, 05:09 |
| HLA-DQB1 | 16 | 14 | 03:04, 05:04 |
| HLA-DRB1 | 36 | 19 | 03:02, 04:02, 04:03, 04:05, 04:06, 04:08, 04:10, 08:03, 08:04, 09:01, 11:02, 11:04, 12:02,  14:04, 14:06, 15:03, 16:02 |

Supplementary Table 2. Number of HLA alleles identified in the CLOZUK3 sample, and those remaining after thresholding based on a Minor Allele Frequency of 1% (MAF=0.01).

### Supplementary Table 3a – Directed Acyclic Graph nodes

| **# (Node)** | **Nodes** | **Characteristic** |
| --- | --- | --- |
| 1 | Absolute Neutrophil Count (ANC) | Outcome |
| 2 | Age | Measured |
| 3 | Caffeine Use | Latent |
| 4 | Cigarette Use | Latent |
| 5 | Clozapine Dose | Exposure |
| 6 | Concomitant Medication | Latent |
| 7 | CYP1A2 Activity | Latent |
| 8 | Genetic Predictors of CYP1A2 Activity (CYP1A2 Activity Score) | Measured |
| 9 | Duffy-Null Genotype | Measured |
| 10 | Inflammation (CRP) | Latent |
| 11 | Metabolic Ratio (Clozapine/Norclozapine) | Measured |
| 12 | Nitrenium ion | Latent |
| 13 | Pharmacogenomic (PGx) SNPs | Measured |
| 14 | Plasma Clozapine Concentration ([Cloz]) | Measured |
| 15 | Plasma Norclozapine Concentration ([Norcloz]) | Measured |
| 16 | Polygenic Scores (PGS) for Clozapine Metabolism | Measured |
| 17 | Polygenic Scores (PGS) for Norclozapine Metabolism | Measured |
| 18 | Sex | Measured |
| 19 | Time Between Dose and Sample (TDS) | Measured |
| 20 | Treatment Resistant Schizophrenia (TRS) Diagnosis | Measured |
| 21 | Treatment Resistant Schizophrenia (TRS) | Latent |
| 22 | Obesity | Latent |
| 23 | Weight Gain | Latent |

Supplementary Table 3a. Variables Included in DAG showing the causal associations between clozapine dose and absolute neutrophil count in a sample of clozapine users not currently experiencing neutropenia. Table 3a shows DAG nodes and their properties.

### Supplementary Table 3b – Directed Acyclic Graph paths

| **# (Path)** | **From** | **To** | **Summary** |
| --- | --- | --- | --- |
| 1 | Age | Clozapine Dose | Caution is advised when prescribing clozapine to elderly people due to increased likelihood of impaired renal function, resulting in slower titration and/or reduced doses.^31^ |
| 2 | Age | Plasma Clozapine Concentration ([Cloz]) | Age is positively associated with plasma clozapine concentration.^32^ |
| 3 | Age | Plasma Norclozapine Concentration ([Norcloz]) | Age is positively associated with plasma norclozapine concentration.^32^ |
| 4 | Caffeine Use | Absolute Neutrophil Count (ANC) | Caffeine consumption was shown to increase white blood cell count in mice.^33^ |
| 5 | Caffeine Use | CYP1A2 Activity | Caffeine consumption induces CYP1A2 activity.^34^ |
| 6 | Cigarette Use | Absolute Neutrophil Count (ANC) | Cigarette smoking can result in changes to white blood cell count.^35^ |
| 7 | Cigarette Use | Clozapine Dose | Patient smoking status (and smoking cessation) is a key consideration during clozapine titration and dosing.^36^ |
| 8 | Cigarette Use | CYP1A2 Activity | Cigarette smoking induces CYP1A2 activity.^37^ |
| 9 | Cigarette Use | Inflammation (CRP) | Cigarette smoking is associated with increased CRP. ^38^ |
| 10 | Clozapine Dose | Absolute Neutrophil Count (ANC) | Agranulocytosis and Neutropenia are some of the adverse drug reactions that may arise following clozapine use.^39^ |
| 11 | Clozapine Dose | Plasma Clozapine Concentration ([Cloz]) | Increases in clozapine dose generally result in increased plasma clozapine levels.^40^ |
| 12 | Clozapine Dose | Weight Gain | Clozapine use can result in weight gain as an adverse drug reaction; some research has shown that this weight gain also varies with respect to dose.^41^ |
| 13 | Concomitant Medication | Absolute Neutrophil Count (ANC) | Many other medications, not just clozapine, may cause drug-induced neutropenia.^42^ |
| 14 | Concomitant Medication | CYP1A2 Activity | Aside from caffeine and cigarette use, CYP1A2 activity may also be affected by other medications being taken.^43^ |
| 15 | CYP1A2 Activity | Plasma Clozapine Concentration ([Cloz]) | CYP1A2 is involved in the metabolism of clozapine to norclozapine, therefore increased activity results in faster clearance of clozapine, resulting in decreased levels.^44^ |
| 16 | Duffy-Null Genotype | Absolute Neutrophil Count (ANC) | The Duffy-Null Genotype is associated with decreased baseline absolute neutrophil count in carriers compared to non-carriers.^45^ |
| 17 | Genetic Predictors of CYP1A2 Activity (CYP1A2 Activity Score) | CYP1A2 Activity | A pharmacogenomic activity score is a proxy of enzyme activity calculated on the basis of pharmacogenomic variants of known function in a given pharmacogene. While initially developed for CYP2D6, we have applied it to CYP1A2 based on PharmGKB VIP information.^46^ |
| 18 | Inflammation (CRP) | CYP1A2 Activity | Elevated CRP is associated with reduced CYP1A2 activity. ^47^ |
| 19 | Metabolic Ratio (Clozapine/Norclozapine) | Absolute Neutrophil Count (ANC) | Increases in metabolic ratio (increased plasma clozapine with respect to plasma norclozapine) are associated with decreased ANC.^22^ |
| 20 | Nitrenium ion | Absolute Neutrophil Count (ANC) | The nitrenium ion is thought to be a key instigator of neutrophil apoptosis in clozapine users.^48^ |
| 21 | Obesity | Absolute Neutrophil Count (ANC) | Increased weight and obesity have been associated with elevated neutrophil counts.^49^ |
| 22 | Obesity | Clozapine Dose | Clozapine clearance may be slower in obese individuals therefore clozapine dose may be adjusted accordingly.^50,51^ |
| 23 | Obesity | Inflammation (CRP) | Obesity is associated with increased levels of CRP.^52^ |
| 24 | PGS for Clozapine Metabolism | Plasma Clozapine Concentration ([Cloz]) | PGS for clozapine metabolism was associated with plasma clozapine concentration.^4^ |
| 25 | PGS for Norclozapine Metabolism | Plasma Norclozapine Concentration ([Norcloz]) | PGS for norclozapine metabolism was associated with plasma norclozapine concentration.^4^ |
| 26 | Pharmacogenomic (PGx) SNPs | Metabolic Ratio (Clozapine/Norclozapine) | rs1126545_T was associated with increased metabolic ratio.^14^ |
| 27 | Pharmacogenomic (PGx) SNPs | Plasma Clozapine Concentration ([Cloz]) | rs2472297_T was associated with decreased plasma clozapine levels.^14^ |
| 28 | Pharmacogenomic (PGx) SNPs | Plasma Norclozapine Concentration ([Norcloz]) | rs2011425_G and rs61750900_T are both associated with decreased plasma norclozapine levels.^14^ |
| 29 | Plasma Clozapine Concentration ([Cloz]) | Absolute Neutrophil Count (ANC) | Increases in plasma clozapine concentration are associated with decreased ANC.^22^ |
| 30 | Plasma Clozapine Concentration ([Cloz]) | Metabolic Ratio (Clozapine/Norclozapine) | Plasma clozapine concentration is used alongside plasma norclozapine concentration to calculate metabolic ratio.^53^ |
| 31 | Plasma Clozapine Concentration ([Cloz]) | Nitrenium Ion | Clozapine can be oxidised in the presence of hypochlorous acid and chloride to the nitrenium ion.^54^ |
| 32 | Plasma Clozapine Concentration ([Cloz]) | Plasma Norclozapine Concentration ([Norcloz]) | Norclozapine is the primary product of CYP-mediated clozapine metabolism.^55^ |
| 33 | Plasma Norclozapine Concentration ([Norcloz]) | Absolute Neutrophil Count (ANC) | Increases in plasma norclozapine concentration are associated with increased ANC.^22^ |
| 34 | Plasma Norclozapine Concentration ([Norcloz]) | Metabolic Ratio (Clozapine/Norclozapine) | Plasma norclozapine concentration is used alongside plasma clozapine concentration to calculate metabolic ratio.^53^ |
| 35 | Plasma Norclozapine Concentration ([Norcloz]) | Nitrenium Ion | Norclozapine may also react to form a reactive nitrenium intermediate.^56^ |
| 36 | Sex | Absolute Neutrophil Count (ANC) | Being female is associated with increased neutrophil counts in comparison to being male.^57^ |
| 37 | Sex | Clozapine Dose | Patient sex taken into consideration during clozapine titration.^36^ |
| 38 | Sex | CYP1A2 Activity | Being male is associated with increased CYP1A2 activity in comparison to being female. ^58^ |
| 39 | Sex | Plasma Clozapine Concentration ([Cloz]) | Being female is associated with increased plasma clozapine concentration in comparison to males.^59^ |
| 40 | Sex | Treatment Resistant Schizophrenia (TRS) | Men are more likely to develop treatment resistant schizophrenia than women.^60^ |
| 41 | Time Between Dose and Sample (TDS) | Plasma Clozapine Concentration ([Cloz]) | Assays were restricted to when the sample was taken within 6 - 24 hours of the dose as plasma concentrations beyond this range might be confounded.^61^ |
| 42 | Time Between Dose and Sample (TDS) | Plasma Norclozapine Concentration ([Norcloz]) | Assays were restricted to when the sample was taken within 6 - 24 hours of the dose as plasma concentrations beyond this range might be confounded. ^61^ |
| 43 | Treatment Resistant Schizophrenia (TRS) | Treatment Resistant Schizophrenia (TRS) Diagnosis | People with schizophrenia who are not responsive to at least two types of antipsychotic medication, delivered at an appropriate dose, and for an appropriate length of time, and having ruled out non-adherence are diagnosed with Treatment Resistant Schizophrenia.^62^ |
| 45 | Treatment Resistant Schizophrenia (TRS) Diagnosis | Clozapine Dose | Clozapine is prescribed to individuals with a diagnosis of treatment resistant schizophrenia due to its effectiveness at dealing with refractory symptoms of schizophrenia.^63^ |
| 46 | Weight Gain | Absolute Neutrophil Count (ANC) | Increased weight and obesity have been associated with elevated neutrophil counts.^49^ |

Supplementary Table 3b. Variables Included in DAG showing the causal associations between clozapine dose and absolute neutrophil count in a sample of clozapine users not currently experiencing neutropenia. Table 3b shows the paths included in the DAG and their rationale.

### Supplementary Table 4 – Characterising CYP1A2 Activity Score

|  | **Plasma Clozapine Concentration** | | | **Plasma Norclozapine Concentration** | | |
| --- | --- | --- | --- | --- | --- | --- |
| Predictor | Estimate | Std. Error | *p* value | Estimate | Std. Error | *p* value |
| CYP1A2 Activity Score | 0.050 | 0.024 | 0.036 | 0.060 | 0.023 | 0.010 |
| Daily Dose | 0.089 | 0.024 | 2.03 × 10^-4^ | 0.144 | 0.023 | 1.33 × 10^-9^ |
| TDS | -0.028 | 0.024 | 0.248 | -0.011 | 0.023 | 0.646 |
| Sex (Male) | -0.138 | 0.052 | 0.008 | -0.176 | 0.051 | 6.02 × 10^-4^ |
| Age | 0.025 | 0.025 | 0.308 | 0.018 | 0.024 | 0.455 |
| Age^2^ | -0.039 | 0.025 | 0.112 | -0.009 | 0.024 | 0.710 |
| N | 523 | | | 523 | | |

Supplementary Table 4. Results of two generalised linear models testing the association between CYP1A2 activity score with plasma concentrations of clozapine and norclozapine. Standardised regression coefficients are reported alongside standard error and p values. TDS = Time between Dose and Sample

### Supplementary Table 5 – CYP1A2 Activity Score + rs2472297 regression output

|  | **Activity Score Alone** | | | **Activity Score + rs2472297** | | |
| --- | --- | --- | --- | --- | --- | --- |
| Predictor | Estimate | Std. Error | *p* value | Estimate | Std. Error | *p* value |
| Daily Dose | 0.094 | 0.043 | 0.028 | 0.098 | 0.043 | 0.022 |
| [Clozapine] | -0.152 | 0.063 | 0.015 | -0.151 | 0.063 | 0.0159 |
| [Norclozapine] | 0.221 | 0.064 | 5.6 x 10^-4^ | 0.219 | 0.064 | 6.23 x 10^-4^ |
| TDS | 0.054 | 0.029 | 0.057 | 0.056 | 0.029 | 0.05 |
| Sex (Male) | -0.09 | 0.112 | 0.42 | -0.093 | 0.113 | 0.408 |
| Age | 0.213 | 0.051 | 3.5 x 10^-5^ | 0.212 | 0.051 | 4.02 x 10^-5^ |
| Age^2^ | -0.069 | 0.048 | 0.15 | -0.069 | 0.048 | 0.156 |
| CYP1A2 Activity Score | 0.145 | 0.05 | 0.004 | 0.142 | 0.056 | 0.011 |
| rs2472297 |  |  |  | 0.004 | 0.057 | 0.949 |
| Random effects | | | | | | |
| σ^2^ | 0.714 | | | 0.711 | | |
| τ_00_ _LUIN_ | 0.962 | | | 0.969 | | |
| ICC | 0.574 | | | 0.577 | | |
| N _LUIN_ | 523 | | | 521 | | |
|  | | | | | | |
| Observations | 1586 | | | 1580 | | |
| Marginal R^2^ / Conditional R^2^ | 0.058 / 0.599 | | | 0.057 / 0.601 | | |

Supplementary Table 5. Results of two linear mixed effect models testing the impact of rs2472297 inclusion on PGx associations with Absolute Neutrophil Count. Standardised regression coefficients are reported alongside standard error and p values estimated using the *lmerTest* package. TDS = Time between Dose and Sample; ANC = Absolute Neutrophil Count; LUIN = Participant Identifier used in CLOZUK3; ICC = Intraclass Correlation Coefficient.

### Supplementary Table 6 – Duffy Null and PGS regression output

|  | **Duffy-Null Genotype** | | | **Polygenic Risk Scores (PGS)** | | |
| --- | --- | --- | --- | --- | --- | --- |
| Predictor | Estimate | Std. Error | *p* value | Estimate | Std. Error | *p* value |
| Daily dose | 0.104 | 0.043 | 0.017 | 0.097 | 0.043 | 0.024 |
| [Clozapine] | -0.15 | 0.063 | 0.018 | -0.151 | 0.064 | 0.018 |
| [Norclozapine] | 0.216 | 0.065 | 8.32 × 10^-4^ | 0.226 | 0.065 | 0.001 |
| TDS | 0.065 | 0.029 | 0.027 | 0.052 | 0.029 | 0.067 |
| Sex (Male) | -0.112 | 0.114 | 0.325 | -0.094 | 0.113 | 0.409 |
| Age | 0.216 | 0.052 | 4.41 x 10^-5^ | 0.213 | 0.052 | 4.05 x 10^-5^ |
| Age^2^ | -0.1 | 0.049 | 0.042 | -0.071 | 0.049 | 0.143 |
| Duffy-null (Present) | -0.77 | 0.251 | 0.002 |  |  |  |
| Clozapine Metabolism PGS |  |  |  | 0.034 | 0.055 | 0.532 |
| Norclozapine Metabolism PGS |  |  |  | -0.009 | 0.054 | 0.863 |
| Random Effects | | | | | | |
| σ^2^ | 0.713 | | | 0.715 | | |
|  |  |  |  |  |  |  |
| t_00_ _LUIN_ | 0.943 | | | 0.981 | | |
| ICC | 0.569 | | | 0.578 | | |
| N _LUIN_ | 496 | | | 523 | | |
|  | | | | | | |
| Observations | 1526 | | | 1586 | | |
| Marginal R^2^ / Conditional R^2^ | 0.070 / 0.599 | | | 0.046 / 0.598 | | |

Supplementary Table 6. Results of two linear mixed effect models including genetic predictors of Absolute Neutrophil Count. Standardised regression coefficients reported alongside standard error and p values estimated using the *lmerTest* package. TDS = Time between Dose and Sample; ANC = Absolute Neutrophil Count; PGS = Polygenic Score; LUIN = Participant Identifier used in CLOZUK3; ICC = Intraclass Correlation Coefficient.

### Supplementary Table 7 – Condensed HLA allele regression output

| **Gene** | **HLA Allele** | **Estimate** | **Std Error** | ***p* value** | ***p* value (FDR)** | ***p* value (Bonferroni)** |
| --- | --- | --- | --- | --- | --- | --- |
| A | 01:01 | -0.006 | 0.029 | 0.827 | 0.923 | 1 |
| A | 02:01 | -0.006 | 0.031 | 0.856 | 0.928 | 1 |
| A | 02:05 | -0.011 | 0.031 | 0.715 | 0.923 | 1 |
| A | 03:01 | 0.011 | 0.032 | 0.719 | 0.923 | 1 |
| A | 11:01 | 0.03 | 0.031 | 0.335 | 0.923 | 1 |
| A | 23:01 | -0.026 | 0.031 | 0.409 | 0.923 | 1 |
| A | 24:02 | -0.022 | 0.028 | 0.428 | 0.923 | 1 |
| A | 25:01 | 0.005 | 0.031 | 0.865 | 0.928 | 1 |
| A | 26:01 | -0.015 | 0.028 | 0.606 | 0.923 | 1 |
| A | 29:02 | -0.001 | 0.031 | 0.98 | 0.98 | 1 |
| A | 30:01 | -0.008 | 0.029 | 0.783 | 0.923 | 1 |
| A | 30:02 | 0.03 | 0.03 | 0.315 | 0.923 | 1 |
| A | 31:01 | 0.02 | 0.031 | 0.531 | 0.923 | 1 |
| A | 32:01 | 0.007 | 0.028 | 0.814 | 0.923 | 1 |
| A | 33:03 | 0.014 | 0.028 | 0.624 | 0.923 | 1 |
| A | 68:01 | 0.022 | 0.029 | 0.437 | 0.923 | 1 |
| A | 68:02 | -0.055 | 0.031 | 0.079 | 0.923 | 1 |
| B | 07:02 | 0.004 | 0.031 | 0.896 | 0.944 | 1 |
| B | 08:01 | 0.008 | 0.028 | 0.775 | 0.923 | 1 |
| B | 13:02 | -0.01 | 0.03 | 0.743 | 0.923 | 1 |
| B | 14:01 | 0.01 | 0.03 | 0.738 | 0.923 | 1 |
| B | 14:02 | 0.02 | 0.032 | 0.535 | 0.923 | 1 |
| B | 15:01 | -0.011 | 0.028 | 0.702 | 0.923 | 1 |
| B | 18:01 | 0.032 | 0.029 | 0.275 | 0.923 | 1 |
| B | 27:05 | 0.041 | 0.028 | 0.146 | 0.923 | 1 |
| B | 35:01 | -0.022 | 0.029 | 0.457 | 0.923 | 1 |
| B | 35:03 | -0.03 | 0.03 | 0.328 | 0.923 | 1 |
| B | 37:01 | 0.012 | 0.03 | 0.698 | 0.923 | 1 |
| B | 38:01 | 0.005 | 0.028 | 0.851 | 0.928 | 1 |
| B | 39:01 | -0.04 | 0.027 | 0.13 | 0.923 | 1 |
| B | 40:01 | 0.015 | 0.028 | 0.6 | 0.923 | 1 |
| B | 44:02 | -0.031 | 0.03 | 0.3 | 0.923 | 1 |
| B | 44:03 | -0.01 | 0.03 | 0.729 | 0.923 | 1 |
| B | 45:01 | 0.015 | 0.03 | 0.62 | 0.923 | 1 |
| B | 49:01 | 0.013 | 0.027 | 0.625 | 0.923 | 1 |
| B | 50:01 | 0.043 | 0.034 | 0.196 | 0.923 | 1 |
| B | 51:01 | -0.023 | 0.028 | 0.407 | 0.923 | 1 |
| B | 52:01 | 0.018 | 0.026 | 0.502 | 0.923 | 1 |
| B | 53:01 | 0.043 | 0.027 | 0.11 | 0.923 | 1 |
| B | 55:01 | -0.009 | 0.029 | 0.761 | 0.923 | 1 |
| B | 57:01 | -0.044 | 0.03 | 0.143 | 0.923 | 1 |
| B | 58:01 | 0.021 | 0.03 | 0.492 | 0.923 | 1 |
| C | 01:02 | 0.032 | 0.028 | 0.251 | 0.923 | 1 |
| C | 02:02 | 0.036 | 0.028 | 0.197 | 0.923 | 1 |
| C | 03:03 | -0.008 | 0.029 | 0.791 | 0.923 | 1 |
| C | 03:04 | 0.006 | 0.028 | 0.818 | 0.923 | 1 |
| C | 04:01 | 0.005 | 0.03 | 0.872 | 0.928 | 1 |
| C | 05:01 | -0.029 | 0.03 | 0.334 | 0.923 | 1 |
| C | 06:02 | -0.022 | 0.031 | 0.481 | 0.923 | 1 |
| C | 07:01 | 0.024 | 0.029 | 0.409 | 0.923 | 1 |
| C | 07:02 | -0.007 | 0.031 | 0.826 | 0.923 | 1 |
| C | 07:04 | 0.011 | 0.029 | 0.7 | 0.923 | 1 |
| C | 08:02 | 0.026 | 0.031 | 0.399 | 0.923 | 1 |
| C | 12:02 | 0.014 | 0.026 | 0.582 | 0.923 | 1 |
| C | 12:03 | -0.022 | 0.03 | 0.47 | 0.923 | 1 |
| C | 14:02 | 0.022 | 0.028 | 0.431 | 0.923 | 1 |
| C | 15:02 | -0.021 | 0.029 | 0.471 | 0.923 | 1 |
| C | 16:01 | -0.013 | 0.03 | 0.653 | 0.923 | 1 |
| C | 17:01 | -0.038 | 0.032 | 0.237 | 0.923 | 1 |
| DPB1 | 01:01 | 0.014 | 0.03 | 0.631 | 0.923 | 1 |
| DPB1 | 02:01 | -0.048 | 0.029 | 0.099 | 0.923 | 1 |
| DPB1 | 03:01 | -0.024 | 0.03 | 0.432 | 0.923 | 1 |
| DPB1 | 04:01 | 0.016 | 0.03 | 0.582 | 0.923 | 1 |
| DPB1 | 04:02 | 0.035 | 0.029 | 0.224 | 0.923 | 1 |
| DPB1 | 05:01 | 0.013 | 0.027 | 0.63 | 0.923 | 1 |
| DPB1 | 06:01 | 0.012 | 0.027 | 0.667 | 0.923 | 1 |
| DPB1 | 10:01 | 0.007 | 0.029 | 0.806 | 0.923 | 1 |
| DPB1 | 11:01 | -0.051 | 0.03 | 0.088 | 0.923 | 1 |
| DPB1 | 13:01 | 0.023 | 0.033 | 0.482 | 0.923 | 1 |
| DPB1 | 14:01 | 0.061 | 0.033 | 0.068 | 0.923 | 1 |
| DPB1 | 104:01 | -0.02 | 0.031 | 0.512 | 0.923 | 1 |
| DPB1 | 17:01 | -0.001 | 0.026 | 0.96 | 0.968 | 1 |
| DQA1 | 01:01 | -0.04 | 0.028 | 0.157 | 0.923 | 1 |
| DQA1 | 01:02 | 0.017 | 0.032 | 0.593 | 0.923 | 1 |
| DQA1 | 01:03 | -0.031 | 0.031 | 0.323 | 0.923 | 1 |
| DQA1 | 01:04 | 0.026 | 0.029 | 0.361 | 0.923 | 1 |
| DQA1 | 01:05 | -0.026 | 0.03 | 0.399 | 0.923 | 1 |
| DQA1 | 02:01 | -0.003 | 0.031 | 0.923 | 0.947 | 1 |
| DQA1 | 03:01 | 0.037 | 0.028 | 0.196 | 0.923 | 1 |
| DQA1 | 03:03 | -0.018 | 0.032 | 0.581 | 0.923 | 1 |
| DQA1 | 04:01 | 0.009 | 0.029 | 0.741 | 0.923 | 1 |
| DQA1 | 05:01 | 0.019 | 0.029 | 0.495 | 0.923 | 1 |
| DQA1 | 05:05 | -0.017 | 0.029 | 0.551 | 0.923 | 1 |
| DQB1 | 02:01 | 0.02 | 0.029 | 0.485 | 0.923 | 1 |
| DQB1 | 02:02 | 0.015 | 0.032 | 0.637 | 0.923 | 1 |
| DQB1 | 03:01 | -0.028 | 0.03 | 0.343 | 0.923 | 1 |
| DQB1 | 03:02 | 0.039 | 0.028 | 0.168 | 0.923 | 1 |
| DQB1 | 03:03 | -0.008 | 0.029 | 0.769 | 0.923 | 1 |
| DQB1 | 04:02 | 0.009 | 0.029 | 0.759 | 0.923 | 1 |
| DQB1 | 05:01 | -0.045 | 0.028 | 0.116 | 0.923 | 1 |
| DQB1 | 05:02 | 0.041 | 0.027 | 0.126 | 0.923 | 1 |
| DQB1 | 05:03 | 0.03 | 0.029 | 0.296 | 0.923 | 1 |
| DQB1 | 06:01 | 0.021 | 0.031 | 0.5 | 0.923 | 1 |
| DQB1 | 06:02 | -0.002 | 0.032 | 0.954 | 0.968 | 1 |
| DQB1 | 06:03 | -0.048 | 0.031 | 0.124 | 0.923 | 1 |
| DQB1 | 06:04 | -0.013 | 0.028 | 0.645 | 0.923 | 1 |
| DQB1 | 06:09 | 0.05 | 0.032 | 0.115 | 0.923 | 1 |
| DRB1 | 01:01 | -0.024 | 0.028 | 0.392 | 0.923 | 1 |
| DRB1 | 01:02 | -0.003 | 0.03 | 0.912 | 0.944 | 1 |
| DRB1 | 01:03 | -0.068 | 0.028 | 0.016 | 0.623 | 1 |
| DRB1 | 03:01 | 0.022 | 0.029 | 0.437 | 0.923 | 1 |
| DRB1 | 04:01 | -0.025 | 0.03 | 0.412 | 0.923 | 1 |
| DRB1 | 04:04 | 0.073 | 0.029 | 0.011 | 0.623 | 1 |
| DRB1 | 04:07 | -0.021 | 0.033 | 0.527 | 0.923 | 1 |
| DRB1 | 07:01 | -0.004 | 0.031 | 0.909 | 0.944 | 1 |
| DRB1 | 08:01 | 0.011 | 0.029 | 0.709 | 0.923 | 1 |
| DRB1 | 10:01 | -0.031 | 0.03 | 0.295 | 0.923 | 1 |
| DRB1 | 11:01 | -0.005 | 0.028 | 0.846 | 0.928 | 1 |
| DRB1 | 12:01 | -0.033 | 0.029 | 0.249 | 0.923 | 1 |
| DRB1 | 13:01 | -0.038 | 0.031 | 0.222 | 0.923 | 1 |
| DRB1 | 13:02 | 0.018 | 0.029 | 0.526 | 0.923 | 1 |
| DRB1 | 13:03 | 0.023 | 0.032 | 0.47 | 0.923 | 1 |
| DRB1 | 14:01 | 0.028 | 0.029 | 0.332 | 0.923 | 1 |
| DRB1 | 15:01 | -0.014 | 0.033 | 0.673 | 0.923 | 1 |
| DRB1 | 15:02 | 0.024 | 0.031 | 0.43 | 0.923 | 1 |
| DRB1 | 16:01 | 0.087 | 0.027 | 0.001 | 0.157 | 0.157 |

Supplementary Table 7. Condensed results of Linear Mixed Models exploring predictors of Absolute Neutrophil Count (ANC) in the CLOZUK3 sample. HLA genotype was included as a predictor alongside pharmacokinetic variables (i.e., daily clozapine dose, plasma clozapine and norclozapine concentration) and other covariates (i.e., TDS, sex, age, age2). Standardised regression coefficients for the effect of HLA genotype from each analysis alone are reported alongside standard error and p values estimated using *lmerTest* package. Bonferroni & FDR-adjusted p values are also reported. HLA = Human Leukocyte Antigen; FDR = False Discovery Rate.

### Supplementary Table 8 – Single Mediator analysis of Clozapine concentration

| **Lavann()** | | | | | **Mediation()** | | | | |
| --- | --- | --- | --- | --- | --- | --- | --- | --- | --- |
| **Output** | **Estimate** | **95% CI Lower** | **95% CI Upper** | ***p* value** | **Output** | **Estimate** | **95% CI Lower** | **95% CI Upper** | ***p* value** |
| Indirect Effect | 0.008 | -0.01 | 0.026 | 0.394 | ACME (Average) | 0.006 | -0.014 | 0.026 | 0.574 |
| Direct Effect | 0.172 | 0.085 | 0.259 | 1.09 × 10^-4^ | ADE (Average) | 0.155 | 0.093 | 0.222 | <2 x 10^-16^ |
| Total Effect | 0.18 | 0.094 | 0.265 | 3.64 × 10^-5^ | Total Effect | 0.161 | 0.099 | 0.226 | <2 x 10^-16^ |
|  |  |  |  |  | Prop. Mediated (Average) | 0.033 | -0.094 | 0.179 | 0.574 |

Supplementary Table 8. Output from single-variable mediation analyses of dose on ANC (Absolute Neutrophil Count) via plasma clozapine concentration. Table shows estimates of average causal mediated effects (ACME; indirect effect), average direct effect (ADE; direct effect), total effect, and proportion mediated from the *lavann* (using cross sectional data) and *meditation* (using longitudinal data) packages.

### Supplementary Table 9 – Single Mediator analysis of Norclozapine concentration

| **Lavann()** | | | | | **Mediation()** | | | | |
| --- | --- | --- | --- | --- | --- | --- | --- | --- | --- |
| **Output** | **Estimate** | **95% CI Lower** | **95% CI Upper** | ***p* value** | **Output** | **Estimate** | **95% CI Lower** | **95% CI Upper** | ***p* value** |
| Indirect Effect | 0.025 | 1.62 × 10^-4^ | 0.05 | 0.049 | ACME (Average) | 0.029 | 0.007 | 0.053 | 0.008 |
| Direct Effect | 0.154 | 0.066 | 0.243 | 6.14 × 10^-4^ | ADE (Average) | 0.133 | 0.066 | 0.204 | <2 x 10^-16^ |
| Total Effect | 0.18 | 0.094 | 0.265 | 3.64 × 10^-5^ | Total Effect | 0.162 | 0.096 | 0.227 | <2 x 10^-16^ |
|  |  |  |  |  | Prop Mediated (Average) | 0.181 | 0.045 | 0.387 | 0.008 |

Supplementary Table 9. Output from single-variable mediation analyses of dose on ANC (Absolute Neutrophil Count) via plasma norclozapine concentration. Table shows estimates of average causal mediated effects (ACME; indirect effect), average direct effect (ADE; direct effect), total effect, and proportion mediated from the lavann (using cross sectional data) and meditation (using longitudinal data) packages.

### Supplementary Table 10 – Replication regression outputs

|  | **Model 1** | | | **Model 2** | | | **Model 3** | | |
| --- | --- | --- | --- | --- | --- | --- | --- | --- | --- |
| Predictor | Estimate | Std. Error | *p* value | Estimate | Std. Error | *p* value | Estimate | Std. Error | *p* value |
| Daily dose | 0.126 | 0.046 | 0.006 | 0.127 | 0.046 | 0.006 | 0.073 | 0.059 | 0.215 |
| [Clozapine] | -0.121 | 0.084 | 0.149 | -0.052 | 0.15 | 0.729 | -0.131 | 0.199 | 0.51 |
| [Norclozapine] | 0.208 | 0.085 | 0.015 | 0.139 | 0.151 | 0.358 | 0.275 | 0.2 | 0.169 |
| TDS | -0.006 | 0.044 | 0.897 | -0.008 | 0.044 | 0.863 | 0.013 | 0.057 | 0.816 |
| Sex (Male) | -0.066 | 0.099 | 0.507 | -0.065 | 0.099 | 0.511 | 0.001 | 0.124 | 0.993 |
| Age | 0.27 | 0.047 | 1.24 x 10^-8^ | 0.272 | 0.047 | 1.09 x 10^-8^ | 0.27 | 0.059 | 5.05 x 10^-6^ |
| Age^2^ | -0.062 | 0.047 | 0.186 | -0.061 | 0.047 | 0.198 | -0.086 | 0.058 | 0.14 |
| Metabolic Ratio |  |  |  | -0.045 | 0.081 | 0.581 | 0.033 | 0.216 | 0.878 |
| rs2472297_T |  |  |  |  |  |  | 0.023 | 0.056 | 0.686 |
| rs61750900_T |  |  |  |  |  |  | 0.067 | 0.057 | 0.245 |
| rs2011425_G |  |  |  |  |  |  | -0.068 | 0.056 | 0.224 |
| rs1126545_T |  |  |  |  |  |  | -0.007 | 0.057 | 0.907 |
| N | 811 | | | 811 | | | 517 | | |
| R^2^ / Adjusted R^2^ | 0.066 / 0.058 | | | 0.067 / 0.057 | | | 0.071 / 0.049 | | |

Supplementary Table 10. Replication Analysis: Predictors of Lowest Absolute Neutrophil Count (ANC). Standardised regression coefficients are reported alongside standard error and p values. In line with past work^18^, Model 1 contains pharmacokinetic predictors of ANC, Model 2 additionally includes the Metabolic Ratio (clozapine/norclozapine ratio) covariate. Finally, Model 3 contains both pharmacokinetic and pharmacogenomic covariates as predictors of ANC. TDS = Time between Dose and Sample; ANC = Absolute Neutrophil Count.

## Supplementary References

1. Lynham, A. J. *et al.* DRAGON-Data: a platform and protocol for integrating genomic and phenotypic data across large psychiatric cohorts. *BJPsych Open* **9**, e32 (2023).

2. Das, S. *et al.* Next-generation genotype imputation service and methods. *Nat Genet* **48**, 1284–1287 (2016).

3. McCarthy, S. *et al.* A reference panel of 64,976 haplotypes for genotype imputation. *Nat Genet* **48**, 1279–1283 (2016).

4. Pardiñas, A. F. *et al.* Pharmacokinetics and pharmacogenomics of clozapine in an ancestrally diverse sample: a longitudinal analysis and genome-wide association study using UK clinical monitoring data. *The Lancet Psychiatry* **10**, 209–219 (2023).

5. Lee, S., Shin, J.-Y., Kwon, N.-J., Kim, C. & Seo, J.-S. ClinPharmSeq: A targeted sequencing panel for clinical pharmacogenetics implementation. *PLOS ONE* **17**, e0272129 (2022).

6. Van Rossum, G. & Drake, F. L. Python 3 Reference Manual. (2009).

7. Saiz-Rodríguez, M. *et al.* Polymorphisms in CYP1A2, CYP2C9 and ABCB1 affect agomelatine pharmacokinetics. *J Psychopharmacol* **33**, 522–531 (2019).

8. Lesche, D., Mostafa, S., Everall, I., Pantelis, C. & Bousman, C. A. Impact of CYP1A2, CYP2C19, and CYP2D6 genotype- and phenoconversion-predicted enzyme activity on clozapine exposure and symptom severity. *Pharmacogenomics J* **20**, 192–201 (2020).

9. Tian, D., Natesan, S., White, J. R. & Paine, M. F. Effects of Common CYP1A2 Genotypes and Other Key Factors on Intraindividual Variation in the Caffeine Metabolic Ratio: An Exploratory Analysis. *Clin Transl Sci* **12**, 39–46 (2019).

10. Werk, A. N. & Cascorbi, I. Functional Gene Variants of CYP3A4. *Clinical Pharmacology & Therapeutics* **96**, 340–348 (2014).

11. Reisberg, S. *et al.* Translating genotype data of 44,000 biobank participants into clinical pharmacogenetic recommendations: challenges and solutions. *Genetics in Medicine* **21**, 1345–1354 (2019).

12. Neyshaburinezhad, N., Ghasim, H., Rouini, M., Daali, Y. & Ardakani, Y. H. Frequency of Important CYP450 Enzyme Gene Polymorphisms in the Iranian Population in Comparison with Other Major Populations: A Comprehensive Review of the Human Data. *Journal of Personalized Medicine* **11**, 804 (2021).

13. Phan, L. *et al.* ALFA: allele frequency aggregator. *National Center for Biotechnology Information, US National Library of Medicine* **10**, (2020).

14. Pardiñas, A. F. *et al.* Pharmacogenomic Variants and Drug Interactions Identified Through the Genetic Analysis of Clozapine Metabolism. *AJP* **176**, 477–486 (2019).

15. Goldstein, J. I. *et al.* Clozapine-induced agranulocytosis is associated with rare HLA-DQB1 and HLA-B alleles. *Nat Commun* **5**, 4757 (2014).

16. Konte, B. *et al.* HLA-DQB1 6672G>C (rs113332494) is associated with clozapine-induced neutropenia and agranulocytosis in individuals of European ancestry. *Transl Psychiatry* **11**, 214 (2021).

17. Ninomiya, K., Saito, T., Ikeda, M., Iwata, N. & Girardin, F. R. Pharmacogenomic-guided clozapine administration based on HLA-DQB1, HLA-B and SLCO1B3-SLCO1B7 variants: an effectiveness and cost-effectiveness analysis. *Frontiers in Pharmacology* **13**, (2022).

18. Purcell, S. *et al.* PLINK: A Tool Set for Whole-Genome Association and Population-Based Linkage Analyses. *The American Journal of Human Genetics* **81**, 559–575 (2007).

19. Chang, C. C. *et al.* Second-generation PLINK: rising to the challenge of larger and richer datasets. *GigaSci* **4**, 7 (2015).

20. Zheng, X. *et al.* HIBAG—HLA genotype imputation with attribute bagging. *Pharmacogenomics J* **14**, 192–200 (2014).

21. Levin, A. M. *et al.* Association of HLA-DRB1 with Sarcoidosis Susceptibility and Progression in African Americans. *Am J Respir Cell Mol Biol* **53**, 206–216 (2015).

22. Willcocks, I. R. *et al.* Clozapine Metabolism is Associated With Absolute Neutrophil Count in Individuals With Treatment-Resistant Schizophrenia. *Front Pharmacol* **12**, 658734 (2021).

23. Kronmal, R. A. Spurious Correlation and the Fallacy of the Ratio Standard Revisited. *Journal of the Royal Statistical Society. Series A (Statistics in Society)* **156**, 379–392 (1993).

24. Rohrer, J. M. Thinking Clearly About Correlations and Causation: Graphical Causal Models for Observational Data. *Advances in Methods and Practices in Psychological Science* **1**, 27–42 (2018).

25. Bates, D., Mächler, M., Bolker, B. & Walker, S. Fitting Linear Mixed-Effects Models Using lme4. *Journal of Statistical Software* **67**, 1–48 (2015).

26. Kuznetsova, A., Brockhoff, P. B. & Christensen, R. H. B. lmerTest Package: Tests in Linear Mixed Effects Models. *Journal of Statistical Software* **82**, 1–26 (2017).

27. Tingley, D., Yamamoto, T., Hirose, K., Keele, L. & Imai, K. mediation: R Package for Causal Mediation Analysis. *Journal of Statistical Software* **59**, 1–38 (2014).

28. Rosseel, Y. lavaan: An R Package for Structural Equation Modeling. *Journal of Statistical Software* **48**, 1–36 (2012).

29. Vaquero-Baez, M. *et al.* Clozapine and desmethylclozapine: correlation with neutrophils and leucocytes counting in Mexican patients with schizophrenia | BMC Psychiatry | Full Text. *BMC Psychiatry* **19**, (2019).

30. Patil, I. *et al.* datawizard: An R Package for Easy Data Preparation and Statistical Transformations. *Journal of Open Source Software* **7**, 4684 (2022).

31. Kirrane, A., Majumdar, B. & Richman, A. Clozapine use in old age psychiatry. *BJPsych Advances* **24**, 204–211 (2018).

32. Lane, H.-Y. *et al.* Effects of gender and age on plasma levels of clozapine and its metabolites: analyzed by critical statistics. *Journal of Clinical Psychiatry* **60**, 36–40 (1999).

33. Ramanaviciene, A., Acaite, J., Ramanavicius, A. & Ramanavicius, A. Chronic caffeine intake affects lysozyme activity and immune cells in mice. *Journal of Pharmacy and Pharmacology* **56**, 671–676 (2004).

34. Tantcheva-Poór, I., Zaigler, M., Rietbrock, S. & Fuhr, U. Estimation of cytochrome P-450 CYP1A2 activity in 863 healthy Caucasians using a saliva-based caffeine test. *Pharmacogenetics* **9**, 131–144 (1999).

35. Higuchi, T. *et al.* Current cigarette smoking is a reversible cause of elevated white blood cell count: Cross-sectional and longitudinal studies. *Preventive Medicine Reports* **4**, 417–422 (2016).

36. Correll, C. U. *et al.* A Guideline and Checklist for Initiating and Managing Clozapine Treatment in Patients with Treatment-Resistant Schizophrenia. *CNS Drugs* **36**, 659–679 (2022).

37. Hukkanen, J., Jacob, P., Peng, M., Dempsey, D. & Benowitz, N. L. Effect of nicotine on cytochrome P450 1A2 activity. *Br J Clin Pharmacol* **72**, 836–838 (2011).

38. Gallus, S. *et al.* Effect of Tobacco Smoking Cessation on C-Reactive Protein Levels in A Cohort of Low-Dose Computed Tomography Screening Participants. *Sci Rep* **8**, 12908 (2018).

39. Miller, D. D. Review and Management of Clozapine Side Effects. *J Clin Psychiatry* **61**, 18308 (2000).

40. Rajkumar, A. P., Poonkuzhali, B., Kuruvilla, A., Jacob, M. & Jacob, K. S. Clinical predictors of serum clozapine levels in patients with treatment-resistant schizophrenia. *International Clinical Psychopharmacology* **28**, 50 (2013).

41. de Leon, J., Diaz, F. J., Josiassen, R. C., Cooper, T. B. & Simpson, G. M. Weight gain during a double-blind multidosage clozapine study. *Journal of Clinical Psychopharmacology* **27**, 22–27 (2007).

42. Moore, D. C. Drug-induced neutropenia: a focus on rituximab-induced late-onset neutropenia. *Pharmacy and therapeutics* **41**, 765 (2016).

43. Guo, J. *et al.* Metabolism and mechanism of human cytochrome P450 enzyme 1A2. *Current Drug Metabolism* **22**, 40–49 (2021).

44. Alarcan, H. *et al.* Correlation between assessment of cytochrome P450 1A2 activity and enzyme activity scores, and their relation to clozapine exposure. *British Journal of Clinical Pharmacology* (2023).

45. Legge, S. E. *et al.* A genome-wide association study in individuals of African ancestry reveals the importance of the Duffy-null genotype in the assessment of clozapine-related neutropenia. *Mol Psychiatry* **24**, 328–337 (2019).

46. Mukerjee, G. *et al.* User considerations in assessing pharmacogenomic tests and their clinical support tools. *NPJ genomic medicine* **3**, 26 (2018).

47. Lenoir, C., Rollason, V., Desmeules, J. A. & Samer, C. F. Influence of Inflammation on Cytochromes P450 Activity in Adults: A Systematic Review of the Literature. *Front Pharmacol* **12**, 733935 (2021).

48. Williams, D. P., Pirmohamed, M., Naisbitt, D. J., Uetrecht, J. P. & Park, B. K. Induction of metabolism-dependent and-independent neutrophil apoptosis by clozapine. *Molecular pharmacology* **58**, 207–216 (2000).

49. Sanchez-Pino, M. D. *et al.* Increased inflammatory low-density neutrophils in severe obesity and effect of bariatric surgery: Results from case-control and prospective cohort studies. *EBioMedicine* **77**, (2022).

50. Kuzin, M. *et al.* Body mass index as a determinant of clozapine plasma concentrations: A pharmacokinetic-based hypothesis. *J Psychopharmacol* **35**, 273–278 (2021).

51. Reeves, S. *et al.* A population pharmacokinetic model to guide clozapine dose selection, based on age, sex, ethnicity, body weight and smoking status. *British Journal of Clinical Pharmacology* doi:10.1111/bcp.15691.

52. Choi, J., Joseph, L. & Pilote, L. Obesity and C-reactive protein in various populations: a systematic review and meta-analysis. *Obesity Reviews* **14**, 232–244 (2013).

53. Costa-Dookhan, K. A. *et al.* The clozapine to norclozapine ratio: a narrative review of the clinical utility to minimize metabolic risk and enhance clozapine efficacy. *Expert Opinion on Drug Safety* **19**, 43–57 (2020).

54. Ramli, F. F., Ali, A., Syed Hashim, S. A., Kamisah, Y. & Ibrahim, N. Reduction in Absolute Neutrophil Counts in Patient on Clozapine Infected with COVID-19. *International Journal of Environmental Research and Public Health* **18**, 11289 (2021).

55. Pouget, J. G., Shams, T. A., Tiwari, A. K. & Müller, D. J. Pharmacogenetics and outcome with antipsychotic drugs. *Dialogues in Clinical Neuroscience* **16**, 555–566 (2014).

56. Kyllesø, L. *et al.* Metabolite Profiling of Clozapine in Patients Switching Versus Maintaining Treatment. *J Clin Psychopharmacol* **42**, 470–474 (2022).

57. Bain, B. J. & England, J. Normal haematological values: sex difference in neutrophil count. *Br Med J* **1**, 306–309 (1975).

58. Scandlyn, M. J., Stuart, E. C. & Rosengren, R. J. Sex-specific differences in CYP450 isoforms in humans. *Expert Opinion on Drug Metabolism & Toxicology* **4**, 413–424 (2008).

59. Tang, Y. *et al.* Gender, age, smoking behaviour and plasma clozapine concentrations in 193 Chinese inpatients with schizophrenia. *British journal of clinical pharmacology* **64**, 49–56 (2007).

60. Siskind, D. *et al.* Rates of treatment-resistant schizophrenia from first-episode cohorts: systematic review and meta-analysis. *Br J Psychiatry* **220**, 115–120 (2022).

61. Flanagan, R. A practical approach to clozapine therapeutic drug monitoring. *CMHP Bulletin* **2**, 4–5 (2010).

62. Howes, O. D. *et al.* Treatment-Resistant Schizophrenia: Treatment Response and Resistance in Psychosis (TRRIP) Working Group Consensus Guidelines on Diagnosis and Terminology. *AJP* **174**, 216–229 (2017).

63. Meltzer, H. Y. Treatment-resistant schizophrenia-the role of clozapine. *Current medical research and opinion* **14**, 1–20 (1997).
